# Sustainable small-scale fisheries markets in the Mediterranean: weaknesses and opportunities

**DOI:** 10.1007/s40152-021-00222-5

**Published:** 2021-04-29

**Authors:** Jerneja Penca, Alicia Said, Marta Cavallé, Cristina Pita, Simone Libralato

**Affiliations:** 1 Euro-Mediterranean University (EMUNI), Piran, Slovenia; 2 Department of Fisheries and Aquaculture of Malta, Luqa, Malta; 3 Low-Impact Fishers of Europe – LIFE, Barcelona, Spain; 4 International Institute for Environment and Development (IIED), London, UK; 5 CESAM - Centre for Environmental and Marine Studies, Department of Environment and Planning, University of Aveiro, Aveiro, Portugal; 6 National Institute of Oceanography and Applied Geophysics – OGS, Borgo Grotta Gigante, Italy

**Keywords:** Sustainable fisheries, Seafood markets, Fisheries value chains, Innovation and transformation, Sustainable markets

## Abstract

Improved access to markets by small-scale fisheries (SSF), as called by Sustainable Development Goal 14b and other global and Mediterranean policy documents, is impeded by the existing organisation of value chains and market structures, which are typically antagonistic to the nature of SSF. This article analyses the markets in the Mediterranean to map the drivers and feedback loops that keep fisheries in an unsustainable trajectory and reviews the key innovations in support of a socially, economically and environmentally sustainable small-scale fishing sector. We show how the current market is dominated by lack of product traceability and underappreciation of the inherent value of SSF products (e.g. local production, freshness, season dependence, quantitatively and culinary varied nature). In addition, due to a lack of organisation and the capacity to act, small-scale fishers are poised to have little to no influence over the price. In what we conceptualise as a response to the negative effects of existing market structures, we identify and classify initiatives that add value to SSF products, but not exclusively. These are the shortening of the value chain, innovation in the distribution channel, diversification in the type of product offered, promotion and education regarding SSF products, label and brand development and the empowerment of SSF communities through improved leadership, ownership, cooperation and coordination. We provide examples of these activities and propose the key types of intervention at various levels of governance to accelerate and capitalise on them in order to accomplish policy goals and achieve a better status of both the oceans and the fishers.

## Introduction

As fisheries are becoming increasingly embedded in the wider governance agenda at the intersection of many policy demands with a focus on sustainability, responsible fisheries management with and for small-scale fisheries (SSF) is gaining traction. The contribution of fisheries markets to sustainability (e.g. seafood campaigns, ecolabelling) has largely avoided taking specific account of SSF (Penca 2020), and the strengthening of the SSF sector was instead delegated to the policy domain. However, growing literature is exploring markets by SSF as venues of a sustainability transition, alongside regulation of access to fishery resources (Stoll et al. 2015; Bolton et al. 2016; Witter and Stoll 2017; Penca 2019; Duggan et al. 2020). This article contributes to this literature by presenting the dual nature of markets for SSF—as both an obstacle and an emerging opportunity for their empowerment and adjustment.

The features of the fisheries markets represent one of the main factors leading to the intensification of exploitation of fishery resources and the development of such fishing practices that have a direct negative consequence on the health of coastal ecosystems (Smith et al. 2010). For example, low, unstable and uncertain prices for fishers’ products result in the search for ways to compensate losses by catching higher volumes of fish and deploying more fishing effort—which results in negative feedback loops in the long term. Such trends are also a consequence of international trade supplies in local markets, which drive down the prices of SSF products (Crona et al. 2015). At the same time, both local and international markets provide significant opportunities to bring benefits to SSF, as depicted by a number of diverse market-based initiatives emerging in the Mediterranean as well as in some other parts of the world (Stoll et al. 2015; Swartz et al. 2017; Duggan et al. 2020). This article examines the markets in two ways. Firstly, it details the current market system affecting SSF, reflecting a set of economic, governance and attitudinal issues that represent a relevant source of problems for fishers. Secondly, it opens up the ways for leveraging market opportunities, which could empower SSF and, in turn, contribute to global sustainability. While focusing on the markets, we reveal how SSF markets are embedded in, rather than separate from, enduring policies and governance structures that have been detrimental to SSF. However, we are also concerned with specific means, through which markets represent an opportunity for the improvement of weaknesses in the organisation of SSF governance.

We locate small-scale fisheries in the context of a transformation required for sustainability—an overarching goal with considerable long-term weight in fisheries (Jentoft 2018) and a long-term, overarching and legal norm (Bosselman 2017). Our point of departure is the contention that SSF are better aligned with sustainability goals in comparison to industrial fisheries (Pauly 2017; Cohen et al. 2019; Said and Chuenpagdee 2019; Said and MacMillan 2020). SSF are also capable of providing multiple contributions to the other policy imperatives underlying the Sustainable Development Goals (SDGs), such as biodiversity conservation and human rights (Food and Agricultural Organisation (FAO) 2018; Morgera and Ntona 2018). The contribution by SSF to sustainability is recognised explicitly, as one of the targets of SDGs - SDG 14b. Nevertheless, we do not assume that sustainable exploitation is a common feature for all SSF (Lloret et al. 2018) and defend evaluating each individual fishery for its multifaceted contribution. The article’s novel contribution to the literature seeking solutions for healthy, productive and resilient seas lies in providing a nuanced account of the SSF market opportunities and innovative adjustments that come from within SSF itself.

Drawing on a qualitative analysis of SSF activities from across the Mediterranean countries between January 2019 and December 2020, we identify the common patterns of the organisation and functioning of the market. The intention was to understand both the problems of SSF products in the markets and the possibilities for their transformation but also to take stock of the transformation of SSF sustainable markets. Research was based on a scoping exercise of initiatives and in-depth case study research, involving workshop presentations, interviews with key individuals involved in activities and an analysis of available material. Rather than evaluating individual situations or initiatives, we highlight the structural weaknesses underlying markets across the region and show how the rise of diverse tangible actions related to the organisation of the supply chain of SSF is a response to those weaknesses and a support to sustainability goals of food provision from the sea.

The focus of the analysis on the Mediterranean region enables a level of granularity as well as the facilitation of conclusions about the shared challenges. While the details implicated in the organisation of value chains are country-specific (e.g. obligatory or habitual system of sale through an auction or absence of it), and, moreover, local-specific and case-dependent (e.g. buyers’ preferences and willingness to pay a premium to SSF products), our account summarises the experience in various places of the region. The narrative points out that problems of suboptimal organisation of value chains and difficulties with markets for SSF are shared across the Mediterranean. However, the relevance of market-based initiatives from some other parts of the world (Abalobi in South Africa or Local Catch in North America) attests that the challenges facing the SSF markets in the Mediterranean support, rather than defy the experience elsewhere.

The article is structured as follows: the “Status and policy goals of Mediterranean SSF” section outlines the Mediterranean SSF sector in the context of the policy goals to which fisheries are expected to contribute, paying particular attention to the role of the markets as an aspect of SSF governance. The “Market struggles of Mediterranean SSF” section discusses the key challenges to accomplishing those goals through markets, by detailing the creation and operation of the SSF markets in the Mediterranean. The “Market-based innovations to respond to challenges” section presents the instances of responses to the challenges by presenting the key types of innovative actions, underpinned by specific examples of initiatives that were introduced in Mediterranean towns or regions to promote SSF products and markets. The “Conclusion” section discusses the adequate measures for leveraging the ongoing initiatives, which are taking place on local or regional scales, in view of the policy commitments.

## Status and policy goals of Mediterranean SSF

SSF^1^ in the Mediterranean are a significant sector in various aspects (Food and Agricultural Organisation (FAO) 2020). Characteristic of this sector is the small capital and energy involvement of individual households. From an environmental point of view, SSF are usually associated with a kind of fishing that is of a lower-impact nature (using a set of passive gears, which do not cause irreparable impact on the seabed) and seasonally diverse (in terms of species, fishing grounds and gears) (Lloret et al. 2018). Moreover, individual SSF also operate at a lower production scale. The polyvalent nature and seasonality employed in the fishing practices are known to respect the biological and migratory cycles of different species (Battaglia et al. 2010). The selectivity of the gears used also generates low levels of discards representing only 10% of discarded catches in the Mediterranean (Food and Agricultural Organisation (FAO) 2020). Also, SSF generate more revenues per investment, greater catches per litre of fuel consumed and more socio-economic added value for every kilo of fish landed (Jacquet and Pauly 2007). Some ongoing analyses demonstrate that SSF produce lower ecosystem impacts for every landed kilogramme than trawlers using an ecosystem modelling approach (Agnetta et al. 2019). Nevertheless, the assumption of low impacts of SSF on ecosystems cannot be applied generally and specific assessments on the sustainability of exploitation need to be performed for each case, as in the example of clam harvesting in the Venice Lagoon demonstrates (Pranovi et al. 2003; Libralato et al. 2004).

In terms of jobs and the local economy, the SSF sector in the Mediterranean encompasses over 84% of the fishing vessels and 29% of revenue and provides large opportunities for employment (59% of total fisheries employment, with each job at sea estimated to create at least 3 to 5 ancillary jobs ashore) (Food and Agricultural Organisation (FAO) 2020). However, while SSF/polyvalent fisheries dominate in terms of the number of vessels and employees, they represent only 15% of total landings from fisheries. These figures demonstrate the high social value and the low production of SSF and explain how SSF are vulnerable to market pressures. From the point of view of local food sovereignty, Mediterranean SSF provide local supplies of fresh fish on a daily basis, including in remote places, and constitute a source of essential/adequate nutrition to the coastal population. Together with SSF worldwide, they are associated with stewardship ethics towards the protection of species and habitats as well as with livelihoods of people in pre-harvesting, harvesting and post-harvesting stages (McConney et al. 2019). Moreover, SSF is an important reservoir of traditional ecological knowledge and an important asset for the tourism sector (Van de Walle et al. 2015). SSF have played a vital role in the maintenance of coastal communities that constitute the key cultural heritage of the Mediterranean (Raicevich et al. 2018). These characteristics make SSF naturally highly adaptable, which is crucial in mitigating the impacts of ecological and economic changes (Battaglia et al. 2010), although increasing uncertainty about fish stock dynamics remains a major challenge for the sustainability of SSF exploitation. Furthermore, SSF have demonstrated to be highly adaptable to climatic changes, to the changes of species composition and to the establishment/appearance of new species (Mancinelli et al. 2017).

The sustainability of Mediterranean SSF has received specific attention in the past years, through the Medfish4ever roadmap, proposed by the European Commission in 2017, and the Regional Plan of Action for Small-scale Fisheries for 2018–2028 (RPOA). Here, all the Mediterranean countries (not only the EU members) vouched their commitment towards the sustainable development of SSF for the next 10 years and beyond. A specific section of the RPOA is dedicated to markets, putting forward various key elements for the development of the SSF value chain, focusing on improved profitability and viability. The RPOA recognises the important role of institutional arrangements in this direction, including the formation of specific organisations dedicated to market enhancement, product quality and traceability. The political commitment to supporting and strengthening the resilience of the SSF in the region at ministerial level was reiterated in 2021 (Union for the Mediterranean (UfM) 2021).

The market-related provisions of the RPOA are also embedded in the FAO Voluntary Guidelines for Small-Scale Fisheries (2015), which are dedicated towards providing guidance in many spheres, including post-harvesting mechanisms for SSF. The Guidelines call for increased focus on the capacity building of fishing communities, such that they can become better equipped in strategising their position in the mainstream markets, finding and creating new market niches and establishing market production plans. These key guidelines emanating from the RPOA and the SSF Guidelines provide opportune avenues for national and regional governments as well as local organisations to undertake new strategies for SSF markets.

These policy priorities for SSF from within the fisheries regime should be read in parallel with the increasing calls for a sustainable transformation of entire food systems towards providing food security and nutrition without compromising the economic, social and environmental bases of future generations. This policy priority, well-embedded in the SDGs, has found support also from within the EU (European Commission (EC) 2020). The urge for a holistic consideration of the way food is produced, processed, distributed, consumed and disposed of as well as its social impacts has brought to the fore the importance of selective practices and local impacts that many SSF have been associated with. The upcoming sections explore how the existing policy imperatives have been developing in practice in the Mediterranean region.

## Market struggles of Mediterranean SSF

The policy orientation of fisheries’ sustainability, detailed in the previous section, has largely failed to have an impact on the markets. This is mostly because the markets are contingent on the existing organisation of value chains and the marketing system and are related to policies, rather than acting independently from them. In other words, it takes more than a new legislation or a policy document to bring on-the-ground transformation of long-standing market systems, due to lock-in effects and institutional path dependencies that can affect the implementation (Wilson 2014). In this section, we provide a thick description of the current realities of Mediterranean markets and reflect on the potential transitional ruptures in achieving an improved strategy for SSF.

The uniqueness of SSF in comparison to other production systems is not adequately recognised in the markets or supply chains (Pascual-Fernandez et al. 2019). Fish is increasingly seen as a homogenous product differentiated mainly by the form in which it is sold—as fresh, frozen, canned or smoked. Supply chains and consumers are not able to consider small-scale production as a quality service, separate from the industrial, farmed and imported products. Symptomatic of the globalised markets is the wide presence of farmed salmon or imported tuna across the coastal towns of the Mediterranean, which tend to diminish the visibility of local SSF products and their inherent value. Some have responded to such effects by trading SSF products elsewhere to fetch higher value in lucrative markets; however, this is not the case across the Mediterranean, as SSF increasingly face competition by bigger market forces.

The inability of Mediterranean SSF to differentiate their products from those of other fisheries is dependent upon two factors. The first are the policies that have made no effort to treat SSF as worthy of special measures and approaches, giving them some sort of recognition in the market. Indirectly, markets have borne the impact of public policies that have through laws, regulations and market interventions, “mainly focused on increasing productivity and facilitating the development of capital-intensive fisheries with larger and more productive vessels” (Pascual-Fernandez et al. 2019). Part and parcel of the predominant governance paradigm that drove the SSF away from rather than to centre stage is the lack of systematic measures for ensuring the visibility of SSF products in the markets and the organisational aspects of their value chains. The other key factor in explaining the marginalisation of SSF products is consumer preferences, the results of poor public education about the seafood trade and sustainability (McClenachan et al. 2016; Lawley et al. 2019) and marketing campaigns, supported by corporate interests (Gutiérrez and Morgan 2015). We segment the layers of the multifaceted problem in turn, in terms of the weaknesses of the current market.

### Lack of traceability

Products from small-scale fisheries get mixed in the sales process with those from semi-industrial and industrial fishing, aquaculture, imported markets and even recreational fishing or illegal fishing (Pascual-Fernandez et al. 2019). The lack of differentiation of SSF products is a reason for the consumer’s insufficient ability to recognise its quality and explains the consumer’s inability to find and acknowledge an SSF product. In most places, including in those with more sophisticated legal frameworks, the requirements for traceability in supply chains and the labelling of products are too inadequate to allow the consumers to appreciate some of the key qualities of products (Penca 2020). Yet mixing SSF products in the supply chain is often the only choice of the small-scale fishers, and this happens when fishers do not have a nearby or constant market to sell to. Fishers that travel several kilometres every day to the nearest point of first sale incur additional transport costs and time investment and, if they sell at auction, are normally subject to suboptimal selling conditions, such as being the last ones to trade their products.

The inability to distinguish SSF products from the catch of industrial fleets and aquaculture other than by informal means is suboptimal for the consumer, small-scale fishers as well as the fisheries regime. From the point of view of the consumers, they are unable to obtain full information on the product they are buying and make informed choices. From the perspective of fishers, the current market status and demand provide them with very few possibilities to influence the market prices. Due to an absence of more stringent transparency requirements, small-scale fishers are unable to present the fair (and ideally premium) value of their products to the consumer, exert any control or influence over the price and consequently and cannot guarantee price stability. This in turn prevents them from improving their socio-economic conditions and improving the stewardship of the resource. A negative cycle of social and environmental consequences is thus established as a result of insufficient traceability.

### Absence of tools to meet requests of sensitive consumers

The lack of traceability requirements impacts on the absence of any tools to meet the expectations of a segment of consumers who are becoming increasingly sensitive to the ethical issues implicated by global trade. After starting to impact the supply chains of most notable cash commodities (e.g. coffee, cocoa, tea, cotton), the trend of increased awareness began to extend to seafood (McClenachan et al. 2016). Yet, the seafood sector had and continues to have very limited tools available to consumers to assess criteria related to the complexity of sustainability in fisheries (Jacquet and Pauly 2007; Richter et al. 2017; Penca 2020). Awareness-raising campaigns are often too nonspecific. Eco-labelling has a limited utility for consumers that are interested in balancing both the social and environmental impact of the products they purchased. Specifically, the Mediterranean is a grey zone for ecolabelling, including for the largest ecolabelling scheme, the Marine Stewardship Council (MSC), which has only two certified fisheries in the Mediterranean to date (Marine Stewardship Council (MSC) 2021). Mandatory labelling also does not fulfil the purpose. For instance, while in the EU the mandatory labelling requirements are normatively considered to be the most advanced since they include the obligation to indicate the fishing gear, the requirements are weakly implemented. For example, a study in Spain on the compliance with obligatory information display in fishery products (Client Earth 2018) revealed that 70% of the obligatory information is not provided, especially the production method (which was missing in 75% of the products) and fishing gear (which is excluded from 85% of the products). Similarly, results from other countries show that the majority of selling points are not compliant with the regulation, including the key information on fishing gear and fishing area (Minoudi et al. 2020; Giovos et al. 2020). While the fishers must collect complete data and usually do so, the loss of data and information occurs in the post-harvest stage, to the disadvantage of the fisher and the consumer.

### Market dominance on a few established products

Mediterranean seafood markets are often dependent on a few highly visible, popular species. These vary from country to country and depend on the preference for species, such as tuna, hake, seabass, seabream, anchovy, molluscs and shrimp (EUMOFA 2020; Centre for the Promotion of Imports from developing countries (CBI) 2020). The popularity of a narrow range of species in the market reflects the consumer (households’ and restaurants’) demand for stability and predictability, including in size and prize. This causes the markets to adjust by securing such products from production systems that can supply them, such as large-scale fishing or aquaculture, or by importing such products, disregarding seasonality and local production. It is clear that the urge for stability in the market poses challenges to the products of SSF, especially in countries where the markets are small and easily saturated. Stability is rather alien to the small-scale production system, which is characterised by seasonality, irregularity and variety of product. The inability to generate continuity and indeed diversity are seen as poor opportunities for the SSF in the context of current market requests.

### Fishers are the weakest in the value chain

The current market system is underpinned by the unequal distribution of power in the value chain with the fishers carrying a disproportional burden. The fishers have little to no control over pricing and are rarely in a position to influence the price. The fact that producers often do not sell to the final consumer, but instead engage with a wide chain of intermediaries and middlemen (fish brokers, fish processors, agents and retailers), represents one source of the problem. The complex and lengthy value chains make it easier to favour the interests of the buyer. Fishers receive marginal earnings for seafood relative to other actors in the value chain, which can be as low as 10% of the final sales price of the product, while the rest goes to intermediaries (Josupeit 2016; Purcell et al. 2018).

### Lack of dedicated organisations and capacity

SSF are usually poorly organised in the sales process, and they each, rather than collectively, negotiate prices with their clients (such as restaurants). This puts them in the position of price-takers and in competition with each other, resulting in a race within the sector, when there is a prior need to strengthen it as a whole. On top of that, small-scale fishers’ sales agreements with customers are normally verbal and fluctuate on a daily basis and disable fishers from having secure prices for the whole year round, let alone multi-annually. To implement an agreement, each fishing family or individual fisher has to take care of the logistics and infrastructure, instead of having resources to share. That means that each family has to have a van (preferably isothermal, but this is not always the case) and the means to store or process their products. Such fishers usually work long hours for relatively low and very uncertain revenues, which may at times not even cover the costs of fishing and the invisible labour included. In the worst case, lacking access to basic accounting tools, credits, micro-finance and insurance, fishers might enter into debt and have few opportunities to break through this cycle of dependency.

The weaknesses discussed are mutually reinforcing and contribute to the creation of tough and uncertain working conditions for fishers, causing vulnerability and the impoverishment of fishers and their families. This in turn directly causes both a premature abandonment of the activity and makes the profession unattractive to the youth. All these factors contribute to discontinued generational renewal (White 2015) and the reduction of future prospects of SSF coastal communities, including women as an essential actor (Frangoudes et al. 2019). As a result of unstable and low prices, fishers may intensify the fishing effort, including by bending the rules or operating on the margins of the law in order to compensate for the low price with a high volume of catches. This has a depressing effect on prices and, of course, aggravates the pressure on marine resources. Furthermore, the product from illicit fishing is sold on illegal markets, which impacts both on the weakening of institutions and a reduction in the long-term revenues of SSF.

## Market-based innovations to respond to challenges

The negative outlook of markets for SSF outlined above can be reversed through measures aimed at the transformation of how markets operate, such as differentiating SSF products from those coming from the industrial and aquaculture sectors. Any such interventions would start by acknowledging SSF as distinguishable from other fisheries and considering their products as worth being made distinct, rather than mixed in the supply chain. Only if SSF products and their quality are recognised as distinct and containing added value are they likely to be rewarded for their quality. Measures in the direction of making SSF products better identifiable in the market can be developed as policies through the means of regulation as well as marketing, branding and organisational activities by SSF and related stakeholders.

Indeed, with the regulatory frame not providing sufficient diversification of SSF products, a number of dissimilar marketing activities related to branding, marketing and the retailing of SSF products have been observed in various parts of the world, framed as alternative seafood marketing programmes (Duggan et al. 2020; Witter and Stoll 2017) or SSF market empowerment tactics (Penca 2019). The Mediterranean provides one vibrant region where several actions to better distinguish market and sell SSF products have emerged, yet the actions are often very limited in scope. Thus, small-scale fishers have developed improvements in infrastructure (ice carrying, distribution logistics), marketing (use of apps, development of new channels of sale) or organisation (clustering the small-scale fishers to jointly present their products). Below, we present the geographical extent of such action in the Mediterranean, the range of innovations introduced and the key features of good practice.

To gather initiatives, we have deployed a two-pronged approach. First, in the mapping exercise, we sought to identify, through direct approaches to stakeholders, internet search and an open call, all the cases of good practice both within and outside the Mediterranean. The presentation of cases is limited to those which are ongoing or recently concluded. The list is by no means exhaustive, and we predict that there are many more instances of actions available in the region. Through direct engagement of key actors in these initiatives or publicly available information about the initiatives, we sought to understand in particular the drivers for action and the types of measures that were developed in response. We also attempted to understand how these initiatives functioned once they were launched.

The market activities of SSF have contributed to the establishment of a customer base, widening of outreach and the optimisation of the supply chain, and subsequently to the improved profitability and viability of the SSF sector, before ultimately enhancing the social welfare of fishers and their ecological stewardship. As such, they are factors that contribute to improved fisheries management (Food and Agricultural Organisation (FAO) 2019). The changes instigated by SSF market initiatives have so far reported as resulting in strengthening a stronger SSF community identity (Duggan et al. 2020) and empowering the SSF as a stakeholder in policymaking (Penca 2019).

We consider and name these market activities as innovations. They provide novel ways of promoting SSF products and markets or organising value chains that contribute to the kind of transformations that improve the fit between human and ecological systems and the capacity of the former to reap the benefits for well-being (Olsson and Galaz 2012). Innovation in this case is not related to technological innovations driving commercial concerns and economic growth but to applications of new ideas in solving societal problems (Dawson and Daniel 2010) by using existing resources and new immaterial approaches to tackle the social conditions (Howaldt and Schwarz 2010). As typical instances of social innovation, they combine structuralist and agentic factors (Cajaiba-Santana 2014), triggering dynamic change and feedback loops for broader society rather than single sectors (Olsson and Galaz 2012). Table 1 provides information on the innovative initiatives, as per the categorisation outlined.

**Table 1:** List of several initiatives aimed at increasing market opportunities for small-scale fishery products in the Mediterranean

Name of initiative	Location	Focus solely on SSF	Type of innovation						
			Shortening value chain	Distribution channel	Product diversification	Promotion and awareness raising	Generation of brand, label, certification	Leadership or ownership	Cooperation
Local and national initiatives within the Mediterranean									
Concha de la Costa	Malaga, Spain	✓	✓	✓	✗	✗	✓	✗	✗
Pescados con Arte	Almeria, Spain	✓	✓	✗	✓	✓	✗	✓	✗
Peix Nostrum	Eivissa, Spain	✗	✓	✓	✓	✓	✗	✗	✗
El Peix al Plat	Barcelona, Spain	✗	✓	✓	✓	✓	✗	✗	✗
Empesca’t	Catalunya, Spain	✓	✓	✗	✓	✓	✓	✓	✗
Peix de custodia	Catalunya, Spain	✓	✗	✗	✗	✓	✓	✗	✗
Golion	Languedoc Roussillon, France	✓	✓	✗	✓	✗	✓	✓	✗
Association pleine mer	France	✓	✓	✗	✗	✗	✗	✗	✗
Poiscaille	France	✓	✓	✓	✓	✗	✗	✗	✗
Thon rouge de ligne	France	✗	✗	✗	✗	✓	✓	✗	✗
Fishbox	Italy	✗	✓	✗	✗	✗	✗	✗	✗
Fresh Fish Alert	Sicily, Italy	✓	✓	✓	✓	✗	✗	✗	✗
Mare e Salute	Gulf of Trieste, Italy	✗	✗	✗	✗	✓	✗	✗	✗
Fisher’s agreements with HORECA	Istria, Croatia	✓	✓	✓	✗	✗	✗	✓	✓
Pick the Alien, iSea	Greece	✓	✗	✗	✓	✓	✗	✗	✓
Istanbul Birlik Fishery Cooperative	Istanbul, Turkey	✓	✓	✓	✗	✗	✗	✓	✗
Kouti Thalasa	Greece	✓	✓	✓	✓	✗	✗	✗	✗
Fish for tomorrow	Malta	✗	✗	✗	✓	✓	✗	✗	✗
Eat Fresh Fish	Malta	✗	✗	✓	✓	✗	✗	✗	✗
Eat the lionfish initiative	Lebanon	✓	✗	✗	✓	✓	✗	✗	✓
Club Bleu Artisanal	Tunisia	✓	✓	✓	✓	✓	✓	✗	✗
Global initiatives with impact in Mediterranean									
Open Food Network	France and Italy	✗	✓	✓	✓	✗	✗	✗	✓
Slowfood presidia	18 fishery presidia in Mediterranean	✓	✓	✗	✗	✓	✓	✗	✓

Adapted from Penca et al. (2020)

As Table 1 shows, the innovations linked to the different initiatives vary depending on the type of intervention and the response to the market. However, the initiatives can be classified as fulfilling one or more of these innovative mechanisms: (i) shortening of value chain, (ii) a focus on the distribution channel, (iii) the concept of product diversification, (iv) generation of a brand and labelling, (v) innovation through leadership or ownership and (vi) heightened cooperation. Even if the actual initiatives normally incorporate more than one of the following models or types of innovation, the clusters of innovations allow for a more systemic reflection on the types of intervention, also inherently reflecting the underlying drivers. We use examples of initiatives under each category not as a means to provide an exhaustive overview of initiatives but rather to illustrate the description of the innovations.

### Shortening the value chain

Innovation in the shortening of the value chain relates to creating a shorter and more direct link between the producer and the consumer (reducing the number of intermediaries) and ideally reaching the point where the producer is able to serve the final customer directly. The geographical distance between the point of production and the final point of consumption can be shortened as a side effect, but this is not the primary objective. The outcome of a shortened value chain is improved traceability, enhanced communication links between the producers and consumers and usually a good price deal for both parties. The traditional means of selling off the boat or at a fishermen’s market located in marinas or ports remains an effective tool of direct sale, where allowed. Direct sales are permitted in most of the Mediterranean countries, even if they are limited in value in some EU countries, where only catches of up to 50 kg of fish can be sold (EC 1224/2009). While not quite a case of innovation in most instances, recent initiatives have emerged to map these direct marketing points at harbour level to inform and/ promote their utilisation by direct consumers (e.g. PleineMer’s map in France).

Apart from producers’ markets, initiatives that have a longer tradition in agriculture, such as organic vegetable boxes and schemes based on the Community Supported Agriculture Model, are only gradually gaining traction among fisheries. These are taking the form of Community Supported Fisheries, fish box and online trading schemes, where schemes derived from agriculture and fisheries hardly interrelate. Some of these innovations are also linked to use a new distribution channel (as described below). The COVID-19 pandemic pushed innovation in this direction to a degree, with local fishers starting to sell online and deliver their products directly to the consumers through simple social media channels or more elaborated tools, e.g. Hook and Deliver (Malta) and Poiscaille (France). Initiatives can be contractual, involving the use of agreements between SSF and restaurants or hotels based on the purchase of the “catch of the day”.

In the Istria region, Croatia, SSF set up agreements with the HORECA channel in order to decrease their dependence on exports to nearby Italian markets (and prices being dictated by the foreign buyers) and sell more on the local market. Opportunities are provided particularly by hotels and restaurants, which demand high-quality local produce with a steady supply and delivery. In response, 50 fishers in this region are organised in a cooperative, which owns a fish processing plant and a purification and dispatch centre for bivalve molluscs. They process the catch and prepare ready-to-cook products (e.g. fish fillets, gutted and cleaned fish, chucked and cleaned scallops), smoked products as well as processed seafood. The cooperative can also take special orders from hotel and restaurants and delivers the products directly to buyers.

Short supply chains do not necessarily result in less kilometres travelled. Fresh seafood can be sent to where the expected value is higher. Quite often, urban centres provide a better selling point, because of the higher purchasing power. Thus, under the Golion labelling scheme, some seafood caught by small-scale fishers from the Gulf of Lion in France is sent over 700 km away to Paris to supply the high-end restaurants with quality fish. Direct sales by the fishers reduce the middleman costs, securing fairer prices and higher profit margins from their catches.

### Innovation in the distribution channel

Innovation in the distribution channel may improve sales systems by using novel routes of selling the product. To a large extent, this relates to using ICT (Information and Communication Technology) through apps, online platforms or social media channels to offer their products and services. The use of ICT in the SSF sector has been on the slow rise for years but was accelerated considerably by the outbreak of COVID-19 in 2020, triggered by the need to expand the customer base due to the closure of restaurants. Some online markets specialise in SSF products, typically informing the consumers of the daily catch of the artisanal fisher through an online platform or communication system, sometimes even before the landing. Such examples include the project “Fresh Fish Alert”, a mobile application establishing a direct link between fishers and consumers to enable the virtual marketing of SSF products caught within a set of social and environmental guidelines, which taps into the Sicilian market. Another example is the “Fresco y del Mar” in Spain that offers purchases online and also through phone-to-final consumers. In many places, including the coastlines of Lebanon, the online chat app, WhatsApp, is used widely by small-scale fishers to reach the customers effectively. In many *Cofradías* in Spain, fishers have started to sell their products through the online auction sale (every day at 4 pm).

A breakthrough type of innovation in the context of ICT is a mobile app suite called Abalobi. Abalobi includes an electronic catch documentation, traceability platform, a marketplace and an integrated digital transactional system, allowing the fishers to document their fisheries and related vital data as well as sell their catch to the markets directly. Abalobi was developed for South African SSF, but it is currently being adjusted to the needs of SSF in certain countries in the Mediterranean (Albania, Algeria, Italy, Tunisia, Turkey).

### Diversification in the type of product offered

This innovation relates to offering different products to diversify the types of sale, with the activity responding to the concentration of consumption of relatively few species (hake, cod, shrimp, tuna, seabass, farmed salmon, etc.), which can potentially be overexploited and also do not correspond to the reality of the actual local catch in a particular season. Initiatives emerged to create new markets and valorise the market’s lesser-known species, which are an important part of the catch for SSF, have gastronomic value and help to reduce fishing pressure on overexploited species.

Another key driver for this innovation is the increasing presence of non-indigenous species, particularly in the East Mediterranean due to Suez Canal enlargement, climate change and overfishing (Lejeusne et al. 2010). As some of the non-indigenous species can be edible or even considered delicacies (e.g. blue swimmer crab or lionfish), initiatives have been set up to promote their consumption and increase fishing pressure on them by SSF, e.g. Dairies of the Ocean in Lebanon or iSea from Greece. Activities typically encompass awareness raising, production of recipe books and work with chefs to enhance the perception of the culinary value of new species.

A specific approach to ensuring the diversification of produce is through so-called fish boxes or fish baskets. Having originated in North America, they are gradually making their way to the southern parts of Europe. They operate in a small number of countries, for instance, in Greece under the name of Kouti Thalassa (*a box full of sea*), in the Adriatic Sea, Italy, as FishBox or in Gökova Bay in Turkey. This model is particularly appropriate for considering the Mediterranean characteristics of a large variety of species and the high degree of unpredictability of the catch. In a typical arrangement, the consumer agrees to receive a specific quantity of seafood rather than its exact type, and the content will ultimately depend on the catch. This in turn provides customers with “surprise” fish products that they might not have tasted before, fostering awareness and an appetite for underutilised species.

If a broader view of the markets is deployed to encompass not only seafood products but also other services small-scale fishers can perform, a rise of fishing tourism can be noted as a form of multi-use of the marine space (Guedri and Chakour 2015; Depellegrin et al. 2019; Kyvelou and Ierapetritis 2020). Fishing tourism mostly develops, and is promoted, as an alternative income-generating activity for SSF, while it also contributes to the reduction of fishing pressure on the fishing resources.

### Promotion of the products and awareness raising

The promotional and educational activities seek to showcase the quality of SSF products and highlight the specificities of their catch, with the ultimate objective being a widening of the SSF markets and valorisation of their products. These are probably the most widespread of the approaches to SSF empowerment. Undertakings in this group include printed or digital materials and hands-on activities, such as workshops, gastronomic events, festivals and cooking classes, which target different audiences, from chefs to the general public. These initiatives go beyond providing technical and legal information of the products and promote the intrinsic value of the product, teaching how it was caught or how it is to be prepared, while explaining the ecological significance of the marine species and the low-impact nature of small-scale fishers. Such events not only aim to increase consumer exposure and transmit knowledge but also allow a re-connection and interaction between the consumer and the fishers, facilitating a mutual understanding and increase in trust. While these activities are only indirectly “market-based”, they may importantly influence the preference of the buyers and have an impact on the demand as one of the key drivers of the incumbent market factors.

The QuickFish Guide by Fish4tomorrow NGO surveys commonly purchased species in Malta and evaluates their sustainability in the Maltese context in order to provide a concrete recommendation on their purchase. The same NGO, in cooperation with the Mediterranean Culinary Academy, also trains chefs in Malta to prepare local seafood according to old and forgotten culinary traditions and organises gastronomic events that revive old cooking techniques or introduce new types of SSF products and thus contribute to the popularity of SSF products. Apart from tailored events, many promotional activities are organised regularly by a local community or association of fishermen, such as the summer festival called “Barche aperte” (*open boats*) run in the coastal town of Caorle in the Veneto region, Italy. Visitors are welcome to discover the SSF profession first-hand and can purchase their fish directly from fishers. In Almeria, Spain, the Association of Artisanal Fishers of the Cabo de Gata Marine Reserve (Pescartes) designed a programme of activities aimed at raising awareness of lesser known species (so commonly part of the catch by the SSF sector in the Mediterranean) and the cuisine linked to them in order to increase demand and, therefore, the prices of these species.

### Generation of a brand or labelling scheme

The generation of a brand or label is an organisationally sophisticated method of branding and highlighting the qualities of the product. Labelling schemes are usually initiated by fishers or NGOs, but public authorities have shown interest in them too. These initiatives essentially develop a system of marking the product (through tags stuck to the high-value species, such as lobster, grouper, john dory or dentex, or to boxes in the case of more abundant species) in order to signify to the consumer the distinctiveness of the product. The label can communicate the origin of the product (geographical indication), the production system (exclusively small-scale or artisanal techniques), quality and freshness of the products (e.g. fish of the day) and the management efforts of professional fishers in conserving the local environment. Therefore, underlying the visual identity is typically a system of traceability and robust organisational structure to ensure the recognition and functioning of successful labelling schemes. In the majority of cases, the distinctiveness enables the producers to charge a premium on the price, which is reinvested into the community of fishers and the protection of the local (marine) environment.

One well-developed labelling initiative is Peix Nostrum (*our fish*), which marks the product of professional, mostly small-scale fishers from two ports of Ibiza, Spain. The purpose of the Peix Nostrum brand was to ensure fishers’ participation in selling their catch, guaranteeing the traceability of products. Nowadays, 23 species caught by around 80 fishers employing small-scale passive gear and 4 trawlers are sold with the patented label. The label attests to the product’s premium quality, its freshness, traceability and the efforts of professional fishers in applying self-regulation, including respecting fishing closures and the rules of marine protected areas as well as improvements in fishing gear (more selective and less impactful on the ecosystems). In addition, a formal association markets and distributes seafood in a manner that avoids unfair and unnecessary competition, self-organisation and the overexploitation of fishing grounds, while also negotiating the best product prices per season, equal for all its clients. This strategy has allowed fishers to have market stability and certainty in their daily lives.

The other sophisticated labelling initiative is Golion, which marks the products that have been caught by SSF in the Gulf of Lion, France. It was developed in 2014 by the professional small-scale fishers, in partnership with the Occitania Region, and with financing from an external foundation. The Golion trademark’s strategy is to enable the identification, marking and traceability and valorisation of products of around 80 small-scale fishers working in coastal areas and the salted water lagoon. The logo is used to distinguish and visually identify products. The brand has become known to professional buyers in France, mainly in Paris and the Occitania Region. The success of the trademark has led members to try to organise themselves into a more formal business structure, and they have submitted a request to establish themselves as a producer organisation that will be entirely composed of small-scale fishers.

At the national level, the idea of a nation-wide French label for SSF products has been considered (Petit Péché), but not yet applied. There is considerable interest among consumers for a similar national label to be implemented also in other countries (Zander and Feucht 2018). In Tunisia, the Association Blue Club Artisanal has put in place a system, whereby artisanal Tunisian fishermen are providing identifiable, traceable and quality-controlled SSF products to certified restaurants, including to Sicily, Italy. Such a collaboration across national jurisdictions is a very rare example of the transboundary initiative.

### Innovation in leadership or ownership

This innovation relates to changes introduced at managerial level, which can be instrumental in enabling fishers to become price makers instead of remaining price takers. Typical activities encompass support and capacity building of the sector, including enhancing entrepreneurial skills and improved organisation of their commercialisation initiatives. Such activities not only empower the SSF sector as actors of change but also contribute to the creation of fairer prices for SSF products. An important recognition is that market initiatives in favour of SSF do not need to be owned and led by small-scale fishers. Instead, social entrepreneurs or non-profit organisations can take over the role, with fishers being closely engaged and retaining a level of co-ownership and a sense of co-responsibility for the socio-ecological impact of the fisheries markets.

Attempts at creating small-scale fishing producer organisations (such as in the French Mediterranean or Conil in Spain) provide relevant instances of such disruptions. Producer organisations have the potential to play a key role in the transition to the sustainable management of commercialisation structures. However, the introduction of POs for SSF remains weak, predominantly due to the high level of administrative burden associated with their setup and running (Pascual-Fernandez et al. 2019)—a reality experienced in the agriculture sector more broadly (Council of the EU 2014). In practice, national authorities may pose difficult conditions for establishing producer organisations, such as high volumes of landings, which conflict with the nature of SSF.

### Innovation in cooperation and coordination

The final innovation relates to initiatives that seek to coordinate existing efforts, replicate and scale up individual initiatives (outlined above) with the view to creating a stronger voice of this traditional but neglected sector and enhancing their joint impact. Typical innovations at secondary level operate as networks, including as international endeavours. The only example from the region, with active members in the Mediterranean, is the Slow Food network. Slow Food brings together various local actions that subscribe to its “good, clean and fair food” slogan, including seafood, where it is known under its Slow Fish arm. An example from outside the region includes a network of restaurants in Galicia, Spain, called Restauramar, committed to an ethical code that promotes awareness and the capacity building of stakeholders. Another example is the Local Catch Network, made up of fishers, organisers, researchers and consumers from across North America who exists to support healthy fisheries and the communities that depend on them. Apart from capacity building and knowledge exchange, one of the key outputs by Local Catch is a “seafood finder” that connects profiles, information and contacts from around 500 Community Supported Fisheries and Direct Marketing arrangements in North America, while providing general information to consumers on the values and benefits of these types of arrangement.

### Linking market weaknesses and innovations

**Fig. 1:**
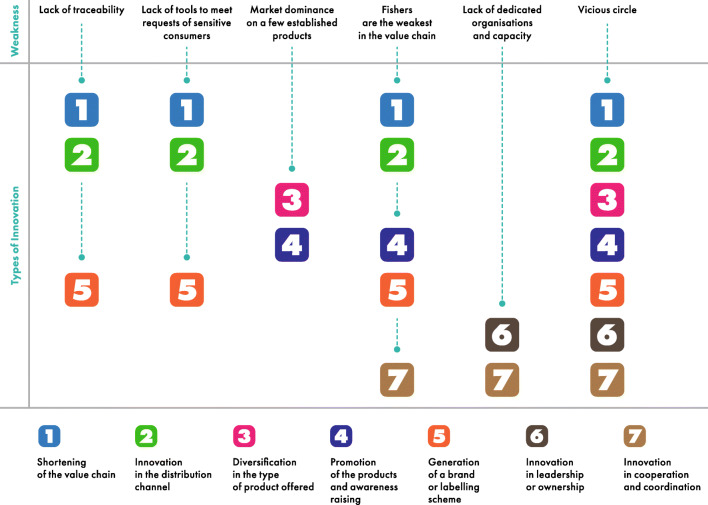
Representation of how different market weaknesses of the SSF sector trigger the types of innovations identified in this work

Weaknesses of SSF markets have triggered specific innovations, which are presented in Fig. 1. The figure points to how certain market weaknesses allow a point of entry for specific innovations. It also demonstrates that just as various weaknesses of the markets for SSF are interrelated, so are the market-based responses. Indeed, most initiatives we surveyed adopt several types of innovation.

The repeated link between the drivers and innovations implies the need for a reflection on the type of region-wide action for scaling up these synergistic activities. This does not represent the urge for a simple replication of existing initiatives or an assumption that there are certain role models for initiatives. The effective market-based initiatives take place starkly on local (and at best regional) scales and are designed in response to local ecological, socio-economic and cultural specificities. The study of SSF market initiatives shows that the establishment of market-based initiatives requires detailed empirical knowledge of the existing struggles and opportunities, needs and circumstances. To scale them up, literature on transitioning to sustainable markets suggests activities in the area of regional exchange of good practices, proving the model(s) and constructing the narrative (Ottosson et al. 2020). These are precisely the elements that currently seem to be missing in the Mediterranean.

Our analysis shows that a lot of market-based initiatives are not focused on SSF only. In part, this reflects the reality of many places where SSF are part of the local fishery industry, which does not represent a homogenous production system. In many instances, the key interest might be on strengthening the competitiveness of *local* supply chains rather than SSF supply chains (Burch and Maes 2017). However, the findings also show that SSF market-based initiatives are part of an ongoing shift towards more conscious food provision, retail and consumption, as part of a social transition to sustainability (Spaargaren et al. 2012; Hinrichs 2014). Apart from some promotional festivals that SSF have traditionally organised in Mediterranean coastal towns, the SSF might in fact be latecomers to the process of market segmentation and the demand for authenticity in food. These processes have come to include SSF, but are not limited to them.

## Conclusion

In order to sidestep multiple obstacles in the regulatory framework (from struggles to ensure fishing opportunities to unfair competition by subsidised industrial fishing), structural and attitudinal factors (that do not stimulate differentiation of SSF products from foreign imports and other production systems) as well as the implications of the weak organisation of the SSF sector, Mediterranean SSF have in recent years developed various approaches to appear in and influence fisheries markets. The actions encompass both local and the targeted (potential) markets and span activities related to branding, marketing, retailing or organisation, jointly using markets to reverse the negative trends related to loss of resources, impoverishment and the gradual disappearance of SSF. In the article, we have used the markets as a narrative to demonstrate the sources of frustration for SSF, opportunities for initiating improved socio-ecological sustainability and the long-term benefits for SSF. We have classified the difficulties of the current SSF markets as well as innovations to respond to them.

We argue that the described market-based activities position small-scale fishers as active agents in steering governance structures towards synergistic sustainability practice at the intersection of environmental, economic, social objectives. As such, they hold promise in the context of the wider transformation required to accomplish the SDGs and sustainability (Cajaiba-Santana 2014; Olsson and Galaz 2012). Additionally, we argue that the presented market-based innovations once again demonstrate the resilience of SSF and their long-term viability (Nayak and Berkes 2019; Pascual-Fernandez et al. 2020; Korda et al. 2021).

Recognising market initiatives developed by SSF in the context of the desirable societal transition and current policy goals provides a justification for their multiplication and scaling-up. To that end, a number of interventions at various levels of governance can take place. In the first place, these encompass the enabling of shared learning and exchange of good practices among existing initiatives as well as encouraging the formation of new initiatives. Structured capacity building plays a key role in both these goals and can encompass training programmes as well as situation-specific, tailored advice. Next, support for SSF market actions should target the provision of local post-harvest infrastructure, launching promotional campaigns for localised, artisanal and small-scale production and deploying adequate fiscal measures (funding, tax incentivisation) to benefit SSF market development.

While promoting positive SSF market actions, attention should be paid to not lowering the standard, for which SSF are associated with responsible practices. It would seem desirable to establish such a standard of good practice as a signpost for action and ensure its wide endorsement. Market-based measures should certainly be coordinated with other policy measures aimed at strengthening responsible SSF, such as those related to improving access to resources of the SSF (Said and MacMillan 2020), removing subsidies harmful to them (Schuhbauer et al. 2020), altering catch documentation schemes and traceability requirements and finally enhancing their enforcement. Particularly now, with the many COVID-19 socio-economic recovery plans taking centre stage both at international and national levels (e.g. the UN Recovering Better 2020 or the EU Green New Deal), it is opportune to discuss the role of scaling up such initiatives to assist in the recuperation of the highly affected SSF markets while shaping their long-term sustainability.

## Footnotes

1 There is no universal definition of what constitutes small-scale fisheries (SSF) in the Mediterranean as these fisheries are heterogeneous across different countries. A definition by the Food and Agricultural Organization of the UN (FAO) classifies small-scale artisanal fisheries as those “involving fishing households (as opposed to commercial companies), using relatively small amount of capital and energy, relatively small fishing vessels (if any), making short fishing trips, close to the shore, mainly for local consumption” (FAO, available at http://www.fao.org/family-farming/detail/fr/c/335263/). The EU used to describe small-scale coastal fishing as the fishing carried out by fishing vessels of an overall length of less than 12 m and not using towed gear (EC 508/2014). The sector is diverse and not easily characterised (Pascual-Fernandez, Pita and Bavinck 2019). In the absence of uniform (and sometimes formal) definitions at the national level, the informal working definition used here is that SSF are characterised in relation to boat length (most commonly, under 12 m), gear type (passive or non-towed gears), technical flexibility (polyvalent gears used according to season and grounds) as well as socio-economic characteristics, such as self-employment and typically remunerated depending on the catch (FAO 2019)
